# Traditional or Modern Contraception? Association Between Health Worker Contact and Contraceptive Choice in India: Findings From NFHS 2019–2021

**DOI:** 10.1111/sifp.70052

**Published:** 2026-04-07

**Authors:** Nandita Bhan, Nicole Johns, Katherine Hay, Vedavati Patwardhan, Abhishek Singh, Shruti Ambast, Lotus McDougal

## Abstract

Despite greater availability and affordability of modern contraception, the use of traditional contraception is rising in India. We examined the relationship between Indian women's contact with a community health worker (CHW) and discussion of family planning (FP) with their contraceptive use. We analyzed data from 306,037 nonpregnant, non‐sterilized married women in the 2019–2021 National Family Health Survey (NFHS). Multinomial regression models estimated the association between CHW contact and contraceptive use (consistent use, switching, and discontinuation) in the past three months. Nearly 22 percent of women reported current use of traditional contraception, with 18 percent reporting exclusive use and 4.1 percent concurrent method use. Traditional contraception was more common among older women, women with lower education, higher parity, and in nuclear households. CHW discussion on FP was associated with higher traditional contraceptive use [Adjusted Odds Ratio (AOR) = 1.11 (95 percent Confidence Interval (CI): 1.04, 1.19)], reversible modern method use [AOR = 1.92 (95 percent CI: 1.82, 2.02)], and concurrent use [AOR = 2.19 (95 percent CI: 1.95, 2.45)]. Recent CHW engagement was associated with consistent modern method use [Adjusted Relative Risk Ratio (ARRR) = 2.02 (95 percent CI: 1.91, 2.13)], switching from traditional to modern method [ARRR = 1.67 (95 percent CI: 1.14, 2.46)], and discontinuation of modern contraception [ARRR = 2.10 (95 percent CI: 1.81, 2.44)]. CHW engagement on FP may enable initiation and consistent use of traditional and modern methods, switching, and discontinuation of contraception.

## INTRODUCTION

Globally, 966 million women of reproductive age use a contraceptive method, the use of which has been associated with fewer unintended pregnancies, more optimal birth intervals, and healthier mothers and children. Modern contraceptive methods (short‐acting, long‐acting, and permanent) form a predominant part of this contraceptive use. Their use has been widely propagated by national family planning (FP) and welfare programs, and research and development efforts have increased the diversity of modern methods available and their effectiveness for pregnancy prevention (WHO 2022). Despite efforts to increase contraceptive choice by making modern contraception accessible and available at lower costs, a significant proportion of women globally (10 percent of contraceptive users) continue to use traditional contraceptive methods, including the withdrawal method (53 million women), the rhythm method (33 million women), and other traditional methods (17 million women) (UNDESA 2022). A majority of these traditional method users reside in low‐ and middle‐income countries (LMICs) (Bertrand, Ross, and Glover 2022).

Despite widely prevalent norms of pronatalism and childbirth as a desired outcome, local cultural knowledge on the menstrual calendar and sexual activity has historically been used by couples to avert unintended pregnancies (Bertrand, Ross, and Glover 2022; Rossier and Corker 2017). Traditional contraceptive methods signify contraceptive knowledge and practices used by communities prior to the advent of modern medicine, which often have cultural, religious, or social origins. The nomenclature of “*traditional*” is used as a parallel to the more recent and “*modern*” methods often associated with allopathic practices and is associated with “*folkloric*” traditions in several communities (Bertrand, Ross, and Glover 2022). However, the measurement of traditional contraception in FP research has widely included the measurement of withdrawal, rhythm, and other context‐specific traditional or folkloric measures. Historical accounts of traditional contraceptive methods have been found in ancient civilizations, including India, and these methods continue to be widely practiced in South Asian and Sub‐Saharan contexts (Bertrand, Ross, and Glover 2022; Rabiu 2018).

The use of traditional methods may appeal to women for a number of reasons, including the lack of reliance on (or connection to) a formal health system, the avoidance of interactions with health workers, and less need for discussion or approval from in‐laws or decision‐making family members (Ajayi, Adeniyi, and Akpan 2018). A qualitative study from the United States on method preferences indicated a desire for flexibility and spontaneity, concerns related to side effects, and satisfaction with methods even if they were less effective (Berglas et al. 2021). These findings are echoed in research across low‐ and middle‐income contexts, where traditional methods are sometimes preferred due to concerns, hesitancy, misinformation, or stigma associated with modern methods (Cohen et al. 2020; Bationo et al. 2022; Bajwa et al. 2012). Traditional methods may also be preferred because they do not involve a product or device placed within one's body, and do not contain hormones, both of which risk breaking social norms and risking the associated sanctions in some communities (Shea et al. 2024). Some traditional methods, such as rhythm, also offer a covert means of FP (Kibira et al. 2020).

Despite this history of the use of traditional contraception, little is known about the networks and people who have passed these traditional methods across generations; this lack of understanding has been further amplified by prevailing norms of secrecy surrounding conversations related to sexual activity, contraception, and the planning of childbirth (Rutenberg and Watkins 1997; Ajayi, Adeniyi, and Akpan 2018). The role of information from sources that include local facilities and community‐based health providers, traditional birth attendants, and locally expert women in the transfer of this traditional knowledge across networks and generations remains largely unexplored.

Community health workers (CHWs), arguably a modern avatar of these transmitters of knowledge, play an important role in spreading knowledge on and access to all contraception, both traditional and modern, primarily in rural areas (Wagner et al. 2018; Sharma et al. 2024). These health workers, tasked with a number of primary health care roles, often have access to women via house‐to‐house outreach and are generally embedded within communities’ social networks. CHWs can often be important agents to dispel misinformation and provide accurate information on the effectiveness of the contraception used (Schaaf et al. 2020; Brooks et al. 2019). This knowledge can also be derived from guidelines such as those by the World Health Organization, which prioritize modern contraception and report lower clinical effectiveness and higher failure rates for traditional methods for pregnancy prevention (WHO 2022). In practice, studies have shown that health providers, including CHWs, can also act as gatekeepers, and their preferences can influence knowledge and use in the community (Soin et al. 2022).

While CHWs have been successful as the backbone of public health service delivery and for developing wider engagement of communities with their health system in some settings, their specific influence on women's contraceptive choice and use is not well understood (Sharma et al. 2024). In some contexts, CHWs are a source of FP‐related information to communities (Schaaf et al. 2020). CHWs can also be engaged in the distribution and the supply chain of getting contraception to communities, thereby ensuring easier access to contraceptive methods and supporting contraceptive use. Emerging research is acknowledging their role in modern contraception provision across LMICs (Brooks et al. 2019), where they provide grassroots delivery channels for the provision of information and services, as well as can also be a reliable and trusted source supportive of family size and planning goals (Scott et al. 2015). In India, CHWs operate in a contraceptive context that was historically influenced by population control priorities and may receive financial incentives for clients who adopt different types of modern contraceptives (Gupte 2017; Department of Health & Family Welfare 2020). In other contexts, there has been mixed evidence on the role of CHWs, where, along with providing services, they may play the role of a “cultural broker” (Schaaf et al. 2020). CHWs have used local music, theatre, or other culturally appropriate approaches to communicate health knowledge and awareness, particularly among minorities or specific populations (Schaaf et al. 2020). A study from Uganda showed that CHW contact and subsequent FP use were greater among older women as discomfort, fear of being judged, and lack of privacy in choices deterred younger women and adolescents from accessing FP through CHWs (Kalyesubula et al. 2021).

In India, an emerging set of studies has reported a rise in the use of traditional contraception over the past decade, with traditional contraceptive method use doubling in 21 of 37 states from NFHS 2015–2016 to NFHS 2019–2021, a trend which has puzzled FP researchers and practitioners (Namasivayam et al. 2023; Ram, Shekhar, and Chowdhury 2014; Halli et al. 2023). Indeed, India's rate of use of traditional methods is the highest in South Asia, concurrent with India also reporting the highest regional rates of overall contraceptive use (ICF International Inc.). In 2019–2021, the prevalence of traditional method use among all currently married women 15–49 years of age was 10.7 percent for any traditional method; 6.1 percent for the rhythm method, 4.6 percent for withdrawal, and less than 0.1 percent for other traditional methods. In India, the rhythm method and withdrawal comprise over 99.9 percent of traditional method use and are discussed and disseminated by CHWs and health providers. India's concurrently rising traditional contraceptive rates, alongside increasing modern contraceptive method use and improvements in women's empowerment indicators, challenge the presumed connection between increased women's agency and greater uptake of modern contraception, in the context of a widening basket of contraceptive options (James‐Hawkins et al. 2018). This complexity operates within a consistent and strong association between women's empowerment indicators and use of modern contraception (Mejía‐Guevara, Cislaghi, and Darmstadt 2021; Rajkhowa and Qaim 2022).

Previous research on the interlinkages between women's empowerment and contraceptive use has several important gaps. First, most analyses have framed traditional and modern contraceptive methods as oppositional alternatives to each other, neglecting to examine the use of traditional contraception in the same analyses as modern contraception, or decisions to switch from traditional to modern methods or vice versa. For instance, studies often examine who is likely to use modern versus traditional contraception (Gebreselassie et al. 2017; Kundu et al. 2022; Nketiah‐Amponsah, Ampaw, and Twumasi Baffour 2022). Many analyses do not consider traditional use even as a primary outcome (Ewerling et al. 2021; Dixit et al. 2021). Second, much existing research has equated agency in FP with modern contraceptive use, rather than positioning agency and choice as important outcomes for FP programming in and of themselves. In doing so, they have often neglected the possibility that women may desire traditional contraception use as a viable contraceptive option, singularly or concurrently, to achieve their fertility needs and desires. Jejeebhoy and Sathar (2024) report that existing measures often exclude the choice for traditional methods as a viable component of the framing for reproductive justice (Jejeebhoy and Sathar 2024). This study builds on this argument by offering concrete evidence of the importance and complexity of considering traditional method use alongside the often‐prioritized modern methods.

In some contexts, the rising use of traditional methods has been associated with male opposition to contraception (Wolff, Blanc, and Ssekamatte‐Ssebuliba 2000); in others, it has been associated with high levels of women's empowerment as measured by decision‐making and beliefs against violence (Castro Lopes et al. 2022). While awareness of traditional methods may be promoted by FP programs, modern methods can often be framed as more desirable due to greater effectiveness, with program implementors sometimes overriding or ignoring women's own preferences without understanding the reason for those preferences (Soin et al. 2022).

In this context, it is important to consider whether there are factors at the level of health systems that explain contraceptive choice and use, to understand the connectedness of women to their health system and the nature of that contact. Understanding the role of health worker contact in contraceptive method use can help ensure that FP programs respect and support women's informed decisions, acknowledge cultural values, and improve overall reproductive health outcomes. Even where the rhythm method, withdrawal, and other methods are mentioned by health providers and workers, FP programs in India, and other LMICs can often prioritize modern contraception in their outreach (Department of Health & Family Welfare 2020; Soin et al. 2022). Hence, the objective of this study was to examine whether contact with CHWs was associated with (1) the type of contraception used and (2) consistent use of, and switching between, traditional versus modern methods.

## METHODS

### Design, Data, and Sample

Our study utilized data from the fifth wave of the National Family Health Survey (NFHS‐5), a nationally representative cross‐sectional survey that was conducted in India from 2019 to 2021. The NFHS is designed and conducted by the International Institute for Population Sciences (IIPS) in Mumbai, India, and is the largest national resource for data on sexual and reproductive health, nutrition, and other key health and development issues with relevance for policy planning and evaluation for women and men of reproductive age. Data for the NFHS were collected using a multistage stratified sampling design, and estimates using the survey are representative at the national, state, and district levels. In this study, we focused our analysis on data from the women's questionnaire, obtained from all eligible women aged 15–49 years in surveyed households.

NFHS‐5 included data from 724,115 women in the age group of 15–49 years, with a response rate of 97 percent. In this study, we limited our analysis to 512,408 currently married women (71 percent of all surveyed women) and further excluded all currently pregnant women and sterilized women at the time of the survey, as our study focused on nonpermanent contraception use. Among married women, 28,241 (6 percent) were pregnant and additional 178,130 (36 percent) were currently sterilized. The final primary analytic sample for the study included 306,037 women who were neither pregnant nor sterilized at the time of the survey.

### Ethical Review

The NFHS‐5 survey was reviewed and approved by the ethical review boards of the IIPS, India, and ICF USA. This analysis utilized publicly available de‐identified data and received a determination of Not Human Subjects Research from the Institutional Review Board of the University of California, San Diego, California.

### Measures

#### Independent Variable

The primary predictor of interest in the study was contact with the CHW in the past three months. The measure was constructed using two items asking whether a respondent had met with an auxiliary nurse midwife (ANM) or lady health visitor (LHV) in the last three months and if they had met with an *anganwadi* worker, accredited social health activist (ASHA), or other CHW in the last three months. We coded response as “yes” if the response was positive to either item, while a “no” response to both was considered no contact.

As a second analysis, we separately examined the two items to assess differences in association by health worker category and to examine whether there was discussion on FP during contact with the CHW. Specifically, we define a binary variable that takes on the value of “yes” if a woman was visited by a CHW in the past three months and also received a FP discussion during that visit, and “no” otherwise. This would imply that women who are classified as “no” include those who (1) were not visited by a CHW in the last three months or (2) were visited by a CHW in the last three months, but who did not receive a FP discussion as part of the visit.

#### Dependent Variables

Since the main focus of our study was on contraceptive use by method type, we were primarily interested in assessing traditional and modern contraceptive use. Current contraceptive use was assessed via the question “*Are you currently doing something or using any method to delay or avoid getting pregnant? [If yes:] Which method are you using*,” with 16 possible responses; respondents could select multiple methods. Traditional methods listed were rhythm, withdrawal, and “other traditional methods.” Modern methods were female and male sterilization, as well as modern spacing methods, namely pill, intrauterine device, male condom, female condom, injectable, diaphragm, foam or jelly, emergency contraception, lactational amenorrhea (LAM), standard days method (SDM), and “other modern method.” The time frame for “current” use is not explicitly defined in the survey, and selecting multiple methods could therefore indicate the simultaneous use of multiple methods in a single sex act or the separate use of multiple methods within the respondent's perception of the “current” time frame, though not simultaneously. We define concurrent use as self‐reported current use of one type of modern and one type of traditional method of contraception (and not concurrent use of any two methods within the same grouping, e.g., two modern methods) in the select‐all survey item noted above. Current reported contraceptive use was consistent with calendar data, with the exception of 302 women (0.06 percent of the sample) who had a birth or termination in the current month but who reported contraceptive use at the time of the survey.

We first constructed a *binary measure of the current use of a traditional method*. For this measure, the traditional method use was indicated if a woman indicated use of rhythm, withdrawal, or “other traditional method” and included women who concurrently used a modern method. Responses were coded as No (the reference group) or Yes. We then constructed *a categorical measure of current use of contraception*, which included four categories: no contraceptive use (reference), modern method use only, traditional method use only, or concurrent use of both a modern and traditional method.

We assessed change in contraceptive use via the reproductive calendar in the survey, which collected information on contraceptive use, pregnancy, termination, and birth on a month‐by‐month basis for the five years prior to the survey. These changes were assessed for the time period of three months prior to the survey using the reproductive calendar data so as to match the time period of the main predictor variable. The DHS methodology for implementing the contraceptive calendar allows for only one contraceptive method to be noted per month; when multiple methods were used by women concurrently, the most effective method was noted. As only one contraceptive method could be listed per month in the contraceptive calendar and concurrent use could not be captured, estimates of current use produced from calendar data differ slightly from estimates produced by the current use measures described above. The contraceptive calendar provided the same list of response options as current contraceptive use, with the exception of emergency contraception or SDM, which were not provided as response options.

We constructed *a categorical measure to assess change in contraceptive use* in the past three months, defined as the change in contraceptive use in the month of survey (current use) and use in the three months prior to the survey. Our measure had nine categories of responses defined as (a) current nonuse of any contraceptive method and nonuse for all three prior months (*consistent* nonuse); (b) current nonuse of contraception following traditional method use (*traditional discontinuation*); (c) current nonuse of contraception following modern method use (*modern discontinuation*); (d) current traditional method use with traditional method use in all three prior months (*consistent traditional use);* (e) current modern method use with modern method use in all three prior months (*consistent modern use*); (f) current traditional method use following one or more months of nonuse within the prior three months (*initiated traditional use*); (g) current modern method use following one or more months of nonuse within the prior three months (*initiated modern use*); (h) current traditional method use following one or more months of modern method use within prior three months (*switched from modern to traditional*); and (i) current modern method use following one or more months of traditional method use within the prior three months (*switched from traditional to modern*). In the cases where a woman had both nonuse and use of the alternate type of method within the prior three months (e.g., used a modern method three months prior to survey, used no method two months prior to survey, and then used a traditional method one month prior to survey and in the survey month) she was coded as “switched.”

#### Covariates

We considered a number of sociodemographic characteristics of women known to be associated with contraceptive use, including women's age (in years), education, household wealth, caste/ethnicity, parity, age at marriage, rural/urban residence, household structure, and husband's age. We categorized education as none, primary (up to five years of schooling), secondary (6–9 years of schooling), or higher (10 or more years of schooling). Household wealth quintiles were used as defined by the NFHS and were constructed on the basis of a household asset index in which households were categorized as poorest, poor, middle, rich or richest, although there has been some concern raised as to the calculation of this measure (e.g., its potential bias towards indicating wealth in urban areas), its use is standard and was retained in the form calculated by and provided within the NFHS microdata (Rutstein 2015; Howe, Hargreaves, and Huttly 2008; Poirier, Grépin, and Grignon 2020; Filmer and Pritchett 2001).

Women's caste/ethnicity status was available from their household data and categorized as per standard constitutional norms of Scheduled Caste (SC), Scheduled Tribe (ST), Other Backward Classes (OBC) versus none/general castes. Parity was measured by the number of living children as reported by women, categorized as 0, 1, 2, 3+. Data were also available on women's age at marriage, which was categorized as less than 18 years versus 18 years or more to indicate child marriage. Residence was categorized as rural versus urban as provided in the NFHS microdata, and household structure was categorized as nonnuclear versus nuclear. Additionally, husband's age was asked for a subsample and was categorized as 15–24, 25–34, 35–44, 45–54, 55+ years, or missing data and used in bivariate analyses.

We constructed a measure for whether a woman's husband was working in the past year, with responses coded as No versus Yes, and for whether a woman's husband was a migrant worker, with responses coded as No versus Yes. However, for both these measures, data were only available for a subset of respondents (*n* = 38,540, 15 percent of the weighted sample). As such, these measures were considered for bivariate analyses but were not included in multivariable models.

#### Analyses

We estimated rates of current traditional and modern contraceptive method use overall, as well as by state and by district, among nonpregnant non‐sterilized women aged 15–49 in India. We examined bivariate comparisons of traditional contraceptive method use rates by all examined sociodemographic characteristics described above, utilizing Pearson's chi‐squared statistics. We also examined the rates of CHW contact, overall and by sociodemographic characteristics, again utilizing Pearson's chi‐squared statistics for bivariate comparisons.

For the regression analyses, we constructed a multivariable multinomial model to assess the association between recent CHW contact and current contraceptive use, using the four‐level current use outcome of no use, modern use only, traditional use only, and concurrent modern and traditional use. We utilized multinomial logistic regression model specifications for unadjusted models and for models adjusted for all covariates (with the exception of the two measures of husband employment due to limited data availability) and state fixed effects. Additionally, we conducted analyses to examine the association between recent contact with CHW with discussion on FP and current contraceptive use, to understand differences in those reporting contact only with those reporting discussion on FP during that contact.

Finally, we constructed a multivariable multinomial model to assess the association between recent CHW contact and past three‐month change in contraceptive use, using the nine‐level change in use outcome described above. We again utilized multinomial logistic regression model specifications for both unadjusted and adjusted regression models, including the same sociodemographic characteristics and state fixed effects. We included an additional analysis to examine the association between CHW contact with discussion on FP with switching or staying consistent with current contraceptive use.

All analyses were conducted using Stata 18.0. All estimates accounted for relevant survey sampling design and weights using the *svy* command; strata with a single primary sampling unit were centered at the grand mean via *singleunit(centered)* specifications for calculation of standard errors.

## RESULTS

Overall, one‐fifth (22.1 percent) of all married nonpregnant and non‐sterilized women in India reported current use of traditional methods, including 13.4 percent rhythm method, 12.2 percent withdrawal method, and < 0.1 percent other traditional methods (note that women could report use of multiple methods). Women could report concurrent use of both modern and traditional methods; 18.0 percent of all married nonpregnant non‐sterilized women reported exclusive use of traditional contraception, and 4.1 percent reported use of both modern and traditional methods. Among all reversible method users (married women using any method of contraception other than female or male sterilization), 44.1 percent used traditional contraception. There was significant variation in traditional and modern method use rates by states (Supporting Information Tables 1 and 2).

Distribution of traditional use by sociodemographic patterns and bivariate associations showed sharp social gradients. Traditional contraception use was higher among older women in the sample (23.0 percent among 25–34 year old and 24.6 percent among 35–49 year old, vs. 13.4 percent among 15–19 year old and 17.1 percent among 20–24 year old, *p* < 0.001), and among those with lower education levels (23.8 percent among women with no education and 23.0 percent among those with primary education vs. 21.8 percent secondary education and 19.8 percent higher education, *p* < 0.001) (Table 1). Minor differences were reported by wealth (range 21.0–22.6 percent, *p* < 0.001). Traditional use was higher among women with higher parity (26.9 percent among 3+ parity and 24.8 percent among parity 2 vs. 8.5 percent parity 0, *p* < 0.001). Traditional use was also higher among women in urban areas (22.8 percent vs 21.7 percent rural, *p* = 0.002) and those living in nuclear households (23.5 percent vs. 20.9 percent nonnuclear households, *p* < 0.001). Traditional use was higher among women who had not had any contact with a CHW in the past three months (22.5 percent vs. 21.4 percent among women who had met a CHW, *p* < 0.001). Among the subset of women for whom husband data were available, women who reported that their husbands were engaged in migrant labor were more likely to use traditional contraception (23.7 percent vs. 19.4 percent, *p* < 0.001). In contrast, modern method users were in the age groups of 20–24 years (31.4 percent) and 25–34 years (38.1 percent) and likely to have secondary or higher education (*p* < 0.001). Modern method users were wealthier, were parity 1 and 2, were more likely to be urban residents, and were more likely to have met a CHW in past three months (*p* < 0.001).

**TABLE 1:** Who is using traditional and modern contraception across states in India? Sociodemographic profile of traditional contraceptive users, nonpregnant women, and non‐sterilized women in the National Family Health Survey – 5 (2019–2021) (*n* = 306,037)

	Traditional method use Number (weighted %)	*p*‐value of chi‐squared test	Reversible modern method use Number (weighted %)	*p*‐value of chi‐squared test
Overall	69,445 (22.1)		10,1746 (23.5)	
Age (in years)				
15–19	1,519 (13.4)	< 0.001	2,397 (23.4)	< 0.001
20–24	9,419 (17.1)		16,368 (31.4)	
25–34	29,948 (23.0)		48,852 (38.1)	
35–49	28,559 (24.6)		34,129 (27.8)	
Education				
None	17,288 (23.8)	< 0.001	18,843 (24.0)	< 0.001
Primary (<5)	9,028 (23.0)		12,513 (31.6)	
Secondary (5–8)	34,029 (21.8)		53,294 (34.6)	
Higher (9+)	9,100 (19.8)		17,096 (39.5)	
Wealth quintile of the household				
Poorest	15,583 (22.6)	< 0.001	18,620 (27.3)	< 0.001
Poorer	15,376 (22.6)		21,134 (30.4)	
Middle	12,850 (21.6)		18,979 (30.3)	
Richer	12,016 (21.0)		19,382 (33.0)	
Richest	13,620 (22.6)		23,631 (40.6)	
Caste/Tribe status				
Scheduled Caste (SC)	12,959 (22.2)	< 0.001	17,966 (31.4)	< 0.001
Scheduled Tribe (ST)	12,015 (20.3)		18,071 (27.3)	
Other Backward Classes (OBC)	25,450 (21.9)		33,643 (29.8)	
None	15,514 (24.2)		23,587 (37.5)	
Don't know	411 (20.7)		487 (25.6)	
Number of living children/ parity				
0	3,330 (8.5)	< 0.001	5,158 (14.4)	< 0.001
1	17,951 (20.7)		29,110 (35.5)	
2	25,124 (24.8)		39,198 (38.9)	
3+	23,040 (26.9)		28,280 (30.5)	
Age at marriage				
<18	23,635 (22.9)	< 0.001	31,091 (30.1)	< 0.001
18+	45,810 (21.7)		70,655 (33.9)	
Area of residence				
Urban	17,895 (22.8)	0.002	28,880 (37.3)	< 0.001
Rural	51,550 (21.7)		72,866 (30.2)	
Husband worked in the past year^a^				
No	1,517 (22.9)	0.8	2,351 (30.2)	< 0.001
Yes	9,301 (22.7)		13,352 (34.5)	
Husband is a migrant worker^a^				
No	8,817 (23.7)	< 0.001	12,731 (35.5)	< 0.001
Yes	2,001 (19.4)		2,972 (28.7)	
Husband age^b^				
15–24	2,858 (17.3)	< 0.001	4,655 (30.0)	< 0.001
25–34	20,813 (21.4)		35,881 (38.2)	
35–44	23,210 (26.6)		34,952 (39.8)	
45–54	13,993 (25.0)		15,103 (25.4)	
55+	1,827 (17.5)		1,802 (15.6)	
Missing	6,744 (14.9)		9,353 (21.4)	
Family structure				
Nuclear	35,505 (23.5)	< 0.001	49,284 (31.9)	< 0.001
Joint	33,940 (20.9)		52,462 (33.3)	
Met CHW in the past three months				
No	43,867 (22.5)	< 0.001	56,915 (29.4)	< 0.001
Yes	25,578 (21.4)		44,831 (37.4)	

^a^ Items asked of only a subset of *n* = 45,953 women (15% of the weighted analytic sample). Items not included in adjusted regression models due to this limited availability.

^b^ Husband age is missing for *n* = 43,164 husbands (16% of the weighted analytic sample) who aren't current members of the household, as husband age is derived from household roster data. Missing age is included as a category in adjusted regression analyses.

Among nonpregnant non‐sterilized women, two in five (39.5 percent) reported meeting a CHW within the prior three months; 24 percent of all women met with an ANM or LHV, and 35.8 percent of all women met with an Anganwadi worker, ASHA, or other CHW. Considering these CHW types jointly, 3.7 percent of all women reported meeting with only an ANM or LHV, 15.4 percent met with only an Anganwadi worker, ASHA, or other CHW, and 20.4 percent met with a CHW from both of those categories. Contact with the CHW was higher among women 20–24 years of age (54.6 percent vs 40.9 percent 15–19, 44.1 percent 25–34, and 26.4 percent 35–49, *p* < 0.001), those with only a secondary level of education (42.6 percent vs. 34.5 percent no education, 39.1 percent primary education, and 38.0 percent higher education, *p* < 0.001), women from the poorest wealth quintile (43.8 percent vs. 43.5–38.0 percent poorer‐richest quintiles, *p* < 0.001), those who belonged to Scheduled Tribe communities (45.1 percent vs. 31.9–42.1 percent for other caste/tribe statuses, *p* < 0.001), parity one women (48.7 percent vs. 22.2 percent among parity zero, *p* < 0.001), and women residing in joint families (43.5 percent vs. 35.0 percent joint families, *p* < 0.001) (Table 2). Rural women also reported a higher likelihood of meeting a CHW in the past three months compared to urban women (43.3 percent vs. 31.7 percent, *p* < 0.001).

**TABLE 2:** Profile of nonpregnant, non‐sterilized women (*n* = 306,037) who reported contact with a community health worker in the past three months in the National Family Health Survey – 5 (2019–2021)

	Met with CHW in the past three months Number (%)	Chi‐squared *p‐value*
Overall	11,8877 (39.5)	
Age (in years)		
15–19	4,284 (40.9)	< 0.001
20–24	28,655 (54.6)	
25–34	55,887 (44.1)	
35–49	30,051 (26.4)	
Education		
None	25,411 (34.5)	< 0.001
Primary	14,583 (39.1)	
Secondary	62,140 (42.6)	
Higher	16,743 (38.0)	
Wealth quintile of the household		
Poorest	28,351 (43.8)	< 0.001
Poorer	27,470 (43.5)	
Middle	23,831 (42.2)	
Richer	21,949 (39.7)	
Richest	17,276 (29.6)	
Caste/tribe status		
SC	23,359 (42.1)	< 0.001
ST	22,725 (45.1)	
OBC	45,579 (40.7)	
None	19,976 (33.6)	
Don't know	583 (31.9)	
Number of living children		
0	8,057 (22.2)	< 0.001
1	40,354 (48.7)	
2	39,184 (40.7)	
3+	31,282 (36.5)	
Age at marriage (in years)		< 0.001
<18	37,502 (37.4)	
18+	81,375 (40.7)	
Area of residence		
Urban	23,790 (31.7)	< 0.001
Rural	95,087 (43.3)	
Husband worked in the past year^a^		
No	2,526 (35.9)	< 0.001
Yes	15,500 (40.7)	
Husband is a migrant worker^a^		< 0.001
No	13,627 (38.8)	
Yes	4,399 (44.0)	
Husband age^b^		< 0.001
15–24	7,882 (48.7)	
25–34	46,538 (50.1)	
35–44	30,189 (35.0)	
44–55	13,705 (25.0)	
55+	2,354 (24.9)	
Missing	18,209 (42.8)	
Family structure		< 0.001
Nuclear	51,066 (35.0)	
Joint	67,811 (43.5)	
Current traditional method use		
No	93,299 (39.9)	< 0.001
Yes	25,578 (38.3)	

^a^ Items asked of only a subset of *n* = 45,953 women (15% of the weighted analytic sample). Items not included in adjusted regression models due to this limited availability.

^b^Husband age is missing for *n* = 43,164 husbands (16% of the weighted analytic sample) who are not current members of the household, as husband age is derived from household roster data. Missing age is included as a category in adjusted regression analyses.

Among women who met a CHW in the preceding three months, one in five (22.3 percent) reported discussion on FP. Among women who reported meeting a CHW, discussion on FP was associated with higher likelihood of use of contraception compared to women who met a CHW but did not discuss FP (49.2 percent vs. 33.5 percent, *p* < 0.001), slightly lower exclusive use of traditional methods (16.3 percent vs. 17.1 percent, *p* = 0.04), higher exclusive use of modern methods (43.5 percent vs. 29.9 percent, *p* < 0.001), and higher concurrent use of modern and traditional methods (6.7 percent vs. 3.9 percent, *p* < 0.001).

In unadjusted models, compared to nonuse of any contraception, recent contact with a health worker was associated with higher odds of use of modern contraception [RRR = 1.46 (95 percent CI: 1.42,1.51)] and concurrent use of both traditional and modern contraception [RRR = 1.35 (95 percent CI: 1.26,1.45)] (Table 3). These associations remained significant after adjusting for covariates and state fixed effects, though the relative risk estimates were attenuated. In adjusted models, compared to nonuse of contraception, recent contact with a health worker was additionally associated with lower use of traditional contraception [ARRR = 0.88 (95 percent CI: 0.85,0.91)] and remained associated with higher use of reversible modern contraceptive methods [ARRR = 1.21 (95 percent CI: 1.17,1.25)] as well as concurrent use of both traditional and modern methods [ARRR = 1.26 (95 percent CI: 1.17,1.35)]. Findings were consistent when examining contact with a specific CHW type (results not shown). Additional analyses to examine the association between CHW contact with discussion on FP (as reported by a subset of women) and current contraception use showed that these women were more likely to use traditional contraception [AOR = 1.11 (95 percent CI: 1.04, 1.19)], modern contraception methods [AOR = 1.92 (95 percent CI: 1.82,2.02)] and concurrent use of both methods [AOR = 2.19 (95 percent CI: 1.95, 2.45)] adjusted for all covariates (Table 4).

**TABLE 3:** Association between recent contact with health worker and current contraception use among nonpregnant, non‐sterilized women in the National Family Health Survey – 5 (2019–2021) (*n* = 306,037)

	Unadjusted			Adjusted for covariates + state FE*		
	Traditional use Ref: No use	Modern use Ref: No use	Concurrent use Ref: No use	Traditional use Ref: No use	Modern use Ref: No use	Concurrent use Ref: No use
	RRR	RRR	RRR	ARRR	ARRR	ARRR
Contact with CHW in the past three months						
Yes	1.02 [0.99,1.06] *p* = 0.18	**1.46**	**1.35**	**0.88**	**1.21**	**1.26**
		**[1.42,1.51]** ***p* < 0.001**	**[1.26,1.45] *p* < 0.001**	**[0.85,0.91] *p* < 0.001**	**[1.17,1.25]** ***p* < 0.001**	**[1.17,1.35]** ***p* < 0.001**

^*^ Models are adjusted for age, education, wealth, caste/tribe, parity, residence, and age at marriage along with state fixed effects in the final models. Bold indicates p<0.05

**TABLE 4:** Association between recent contact with health worker and discussion of FP during most recent contact, and current contraception use among nonpregnant, non‐sterilized women in the National Family Health Survey – 5 (2019–2021) (n = 306,037)

	Unadjusted			Adjusted for covariates + state FE*		
	Traditional use Ref: No use	Modern use Ref: No use	Concurrent use Ref: No use	Traditional use Ref: No use	Modern use Ref: No use	Concurrent use Ref: No use
	RRR	RRR	RRR	ARRR	ARRR	ARRR
FP discussion during contact with CHW in the past three months						
Yes	**1.36** **[1.28,1.45]** ***p* < 0.001**	**2.45** **[2.33,2.57]** ***p* < 0.001**	**2.69** **[2.40,3.02]** ***p* < 0.001**	**1.11** **[1.04,1.19]** ***p* = 0.001**	**1.92** **[1.82,2.02]** ***p* < 0.001**	**2.19** **[1.95,2.45]** ***p* < 0.001**

^*^ Models are adjusted for age, education, wealth, caste/tribe, parity, residence, and age at marriage along with state fixed effects in the final models. Bold indicates p<0.05

We also examined whether recent CHW contact was associated with consistency in use of traditional and modern contraceptive methods over a three‐month period. In unadjusted models, we found that recent CHW contact was associated with higher odds of consistent use of modern methods [RRR = 1.39 (95 percent CI: 1.35,1.44)], higher odds of initiating use both for traditional methods [RRR = 2.40 (95 percent CI: 2.20,2.63)] and modern methods [RRR = 3.35 (95 percent CI: 3.13,3.59)], higher odds of switching, both from modern to traditional methods [RRR = 1.97 (95 percent CI: 1.45,2.68)] from traditional to modern methods [RRR = 2.48 (95 percent CI: 1.81,3.41)], and higher odds of non‐use following discontinuation of modern methods [RRR = 1.99 (95 percent CI: 1.80,2.21)] and traditional methods [RRR = 1.42 (96 percent CI: 1.26,1.59)], relative to consistent non‐use of contraception (Table 5). Adjusting for covariates and state fixed effects modified these associations. We found that in adjusted models, recent CHW contact was associated with a higher likelihood of consistent use of modern contraceptive methods [ARRR = 1.18 (95 percent CI: 1.14,1.22)] but lower likelihood of consistent traditional contraceptive method use [ARRR = 0.86 (95 percent CI: 0.82,0.89)]. Recent CHW contact was associated with a greater likelihood of initiating both traditional and modern methods following non use [traditional initiation ARRR = 1.67 (95 percent CI: 1.51,1.85); modern initiation ARRR = 2.16 (95 percent CI: 2.01,2.33)]. Recent CHW contact was also associated with a greater likelihood of switching from traditional to modern contraceptive method in the period [ARRR = 1.92 (95 percent CI: 1.36,2.73)], while there was no significant association between CHW contact and switching from modern to traditional method use [ARRR = 1.18 (95 percent CI: 0.84,1.63)]. Recent CHW was associated with a greater likelihood of nonuse following discontinuation of a modern method [ARRR = 1.42 (95 percent CI: 1.26,1.59)] but not with nonuse following discontinuation of a traditional method [ARRR = 1.09 (95 percent CI: 0.97,1.24)]. Findings were consistent when examined by contact with a specific CHW type (results not shown). Additional analysis examining the association between CHW contact with FP discussion and consistency in use outcomes (Table 6) that women who reported CHW contact with FP discussion were more likely to report consistent traditional method use [ARRR = 1.13 (95 percent CI: 1.05,1.21)] and consistent modern method use [ARRR = 2.02 (95 percent CI: 1.91,2.13)]], and switching from nonuse to traditional use [ARRR = 1.33 (95 percent CI: 1.14,1.55)] and modern method use [ARRR = 2.08 (95 percent CI: 1.89,2.29)], as well as switching from traditional to modern method use [ARRR = 1.67 (95 percent CI: 1.14,2.46)] and switching from modern to traditional method use [ARRR = 1.64 (95 percent CI: 1.05,2.58)]. CHW contact with FP discussion was also significantly associated with nonuse following discontinuation of a modern method [ARRR = 2.10 (95 percent CI: 1.81,2.44)], but was not associated with nonuse following discontinuation of a traditional method [ARRR = 1.10 (95 percent CI: 0.89,1.35)].

**TABLE 5:** Association (adjusted relative risk ratios, 95 percent confidence intervals, and *p*‐values) between recent contact with health worker and switching or staying consistent with traditional method use among nonpregnant, non‐sterilized women (*n* = 304,381)^a^ in the National Family Health Survey – 5 (2019‐21)

Nonuse, traditional discontinuation	Nonuse, modern discontinuation	Traditional use, consistent	Modern use, consistent	Traditional use, initiated from nonuse	Modern use, initiated from nonuse	Traditional use, switched from the modern method	Modern use, switched from the traditional method
Contact with CHW in the past three months, unadjusted							
**1.43** **[1.28,1.60]** ***p* < 0.001**	**1.99** **[1.80,2.21]** ***p* < 0.001**	1.00 [0.94,1.00] *p* = 0.80	**1.39** **[1.35,1.44]** ***p* < 0.001**	**2.40** **[2.20,2.63]** ***p* < 0.001**	**3.35** **[3.13,3.59]** ***p* < 0.001**	**1.97** **[1.45,2.68]** ***p* < 0.001**	**2.48** **[1.81,3.41]** ***p* < 0.001**
Contact with CHW in the past three months, adjusted for covariates and state FE							
1.09 [0.97,1.24] *p* = 0.14	**1.42** **[1.26,1.59]** ***p* < 0.001**	**0.86** **[0.82,0.89]** ***p* < 0.001**	**1.18** **[1.14,1.22]** ***p* < 0.001**	**1.67** **[1.51,1.85]** ***p* < 0.001**	**2.16** **[2.01,2.33]** ***p* < 0.001**	1.18 [0.86,1.63] *p* = 0.30	**1.92** **[1.36,2.73]** ***p* < 0.001**

**^All =^** All models are adjusted for age, education, wealth, caste/tribe, parity, residence, and age at marriage, along with state fixed effects in the final models. Bold indicates p<0.05

Models exclude women who had a birth or pregnancy termination in the current calendar month.

**TABLE 6:** Association (relative risk ratios, 95 percent confidence intervals, and *p*‐values) between recent contact with a health worker and discussion of FP during most recent contact, and switching or staying consistent with traditional method use among nonpregnant, non‐sterilized women (n = 304,381)^a^ in the National Family Health Survey – 5 (2019‐2021)

nonuse, traditional discontinuation	nonuse, modern discontinuation	Traditional use, consistent	Modern use, consistent	Traditional use, initiated from nonuse	Modern use, initiated from nonuse	Traditional use, switched from the modern method	Modern use, switched from the traditional method
FP discussion during contact with CHW in the past three months, unadjusted							
**1.32** **[1.08,1.62]** ***p* = 0.01**	**2.72** **[2.35,3.14]** ***p* < 0.001**	**1.37** **[1.29,1.46]** ***p* < 0.001**	**2.54** **[2.41,2.67]** ***p* < 0.001**	**1.98** **[1.71,2.30]** ***p* < 0.001**	**3.18** **[2.91‐3.48]** ***p* < 0.001**	**2.42** **[1.55‐3.78]** ***p* < 0.001**	**2.47** **[1.74,3.51]** ***p* < 0.001**
FP discussion during contact with CHW in the past three months, adjusted for covariates and state FE							
1.10 [0.89,1.35] *p* = 0.39	**2.10** **[1.81,2.44]** ***p* < 0.001**	**1.13** **[1.05,1.21]** ***p* = 0.001**	**2.02** **[1.91,2.13]** ***p* < 0.001**	**1.33** **[1.14,1.55]** ***p* < 0.001**	**2.08** **[1.89,2.29]** ***p* < 0.001**	**1.64** **[1.05,2.58]** ***p* = 0.03**	**1.67** **[1.14,2.46]** ***p* = 0.01**

Models exclude women who had a birth or pregnancy termination in the current calendar month. Bold indicates p<0.05

An assessment of the reasons, where available, for switching from traditional method to modern method showed that nearly a third of women reported that they wanted a more effective method (28.2 percent) (Table 7). One in five women (22.8 percent) who switched from traditional to modern methods reported husband disapproval as the reason for switching. Conversely, method‐related reasons, including side effects, inconvenience, lack of privacy, or “did not like method,” were the most common reasons for switching from a modern to a traditional contraceptive method, reported by a quarter of women who switched (26.5 percent). Though one in five women (19.5 percent) who switched from a modern to a traditional method reported wanting a more effective method as the reason for switching, the majority of these women (67.6 percent) were using LAM and likely had either had menses return, ceased exclusive breastfeeding, or passed six months after birth at the time of switch and therefore had to switch to an alternate method.

**TABLE 7:** Reported reasons for discontinuing contraceptive method among women who switched from traditional to modern methods or modern to traditional methods in the past three months in the National Family Health Survey – 5 (2019–2021)

	Reasons for switching from traditional to modern methods (*n* = 610)	Reasons for switching from modern to traditional methods (*n* = 306)
Method failure	**4.3%**	**4.2%**
Became pregnant	4.3%	4.2%
Wanted to become pregnant	**7.7%**	**3.0%**
Wanted to become pregnant	7.7%	3.0%
Method‐related reasons	**10.9%**	**26.5%**
Side effects	1.9%	12.6%
Health concerns	–	–
Inconvenient to use	1.3%	1.6%
Lack of sexual satisfaction	1.5%	1.1%
Creating menstrual problems	0.2%	2.2%
Gained weight	0.1%	0.2%
Did not like method	4.9%	8.2%
Lack of privacy to use	0.9%	0.8%
Wanted a more effective method	**28.2%**	**19.5%**
Wanted a more effective method	28.2%	19.5%
Husband opposed	**22.8%**	**16.1%**
Husband disapproved	22.8%	16.1%
Other fertility‐related reasons	**17.9%**	**15.7%**
Infrequent sex, husband away	17.9%	12.8%
Difficult pregnancy, menopause	–	2.5%
Marital dissolution	–	0.3%
Other non‐fertility‐related reasons	**8.3%**	**15.0%**
Access, availability	2.5%	1.6%
Cost	1.8%	3.6%
Fatalistic	2.5%	5.0%
Other	1.2%	4.4%
Don't know	0.3%	0.3%

Additionally, post hoc analysis to understand state variation by stratifying high prevalence (top quartile) versus low prevalence (bottom quartile) states while replicating adjusted models showed positive and statistically significant associations between CHW contact and nonuse following discontinuation of modern methods as well as initiation of both traditional or modern methods in both high and low traditional use prevalence states, consistent with overall findings (Supporting Information Table 3). The significant negative association between CHW and consistent traditional method use observed overall remained among those states with the highest traditional use prevalence (ARRR = 0.71, 95 percent CI: 0.67,0.75), but this association became null in those states with the lowest traditional method use (ARRR = 1.13, 95 percent CI: 0.94,1.37). Conversely, CHW contact was positively and significantly associated with consistent modern use overall and among low‐traditional use prevalence states (ARRR = 1.46, 95 percent CI 1.32,1.61), but was null in high‐traditional use prevalence states (ARRR = 1.01, 95 percent CI: 0.96,1.07). The associations between CHW and method switching were consistent in high‐traditional use prevalence states, but negligible numbers of method type switchers in low‐prevalence states (*n* = 22 total) precluded estimation of this association in those states.

## DISCUSSION

Our study demonstrated five salient findings. First, we found that one‐fifth of married nonpregnant and non‐sterilized women report current use of traditional methods, which is substantial, particularly in the context of the dominance of female sterilization and the prominent use of reversible modern methods as part of the contraceptive method mix in India. We also note the widespread use of traditional contraception across most Indian states, with 35 of 37 states reporting traditional method use rates of 5 percent or more among non‐sterilized, nonpregnant women, and six states reporting higher traditional method use than modern method use in this population (see Supporting Information Tables 1 and 2).

Second, previous studies have reported that traditional contraception use may be under‐reported (Bertrand, Ross, and Glover 2022; Polis et al. 2016; Bradley, Winfrey, and Croft 2015; Polis et al. 2021); we found that women reported the use of traditional contraception both singularly and concurrently with modern methods. We found associations with lower education, but higher age, and parity, women residing in joint families and in urban areas, demonstrating that, contrary to previous evidence (Gebreselassie et al. 2017; Rossier and Corker 2017), users may be women who consider traditional use as a viable option even while using modern contraception. In one analysis (Gebreselassie et al. 2017), more educated women and those who had completed their family size were more likely to use modern rather than traditional contraception methods. In contrast, another study (Rossier and Corker 2017) showed traditional use to be more common among better educated and higher parity women. Our findings are somewhat contrary to these studies and present a more complex reality, additionally reflective of concurrent use that is masked when concurrent users are labeled with single method use based on efficacy (Croft, Allen, and Zachary 2023).

There is growing research to understand the determinants of traditional use in India and elsewhere, with implications for FP service delivery (Ram, Shekhar, and Chowdhury 2014; Namasivayam et al. 2023; Marquez, Kabamalan, and Laguna 2018; Bertrand, Ross, and Glover 2022). Our findings show that the traditional contraceptive methods are not being used by the most marginalized women—in terms of age or location—and in fact demonstrate complex patterns of use (e.g., reported by women with lower levels of education but who report greater awareness of contraception; women who report husbands are not migrant workers; unclear patterns in the use of traditional contraception by wealth but greater use among general caste groups). This implies the need to replace conceptualizations of traditional methods as substitutes for modern methods with an understanding that they are methods used and relied upon in addition to modern contraceptive methods. Further investigation is needed to understand these complex patterns and to interrogate what these findings on traditional contraception use imply for women's agency in their FP choices.

Third, and interestingly, we found that recent contact with a CHW, which may be a marker of connectedness with the health system, was associated with greater use of modern contraception and concurrent use of both traditional and modern contraceptive methods. We also found that CHW contact was notably associated with consistent use of modern contraceptive methods and higher switching from traditional to modern methods. However, additional analyses indicated that CHW contact combined with FP discussion was associated not only with modern method use or switching from traditional to modern methods, but also led to initiation of both traditional and modern method use, and was associated with higher discontinuation. The association with discontinuation provides greater complexity in our understanding of CHW engagement, not just limiting to the idea of promoting modern methods, but highlighting that perhaps engagement has led to use as well as discontinuation of modern contraception. It is possible that contact with the CHW may have discouraged women from continuing modern method use, especially if side effects remained unaddressed, or encouraged women towards traditional methods. Indeed, our results indicate that method‐related reasons were often the major drivers of discontinuation of modern methods, followed by a desire for a more effective method.

Sensitivity analyses showed that CHW contact that included FP discussion was associated with initiating nonusers to modern and traditional methods both, converting nonusers to users. These findings have two broad implications. One, they indicate the potential role and likely importance of the health system in the uptake of contraception, ensuring its consistency and uptake across methods. CHW engagement was associated with a switch from traditional to modern method use, which may indicate CHW's promotion of modern contraception (which may be attributed to a host of reasons, such as greater effectiveness in pregnancy prevention or any financial incentives associated with some methods). However, we also found that CHW contact was associated with modern method discontinuation, which simultaneously tested this explanation.

Two, findings raise the question whether CHW contact and engagement might, in some circumstances, be associated with women's agency to use contraception, although the directionality of this relationship cannot always be established (Schaaf et al. 2020; Kane et al. 2020). While in our study, we do not have direct measures of agency, previous studies have shown that women's agency has been associated with a higher likelihood of initiating an antenatal care visit and receiving maternal health care services (Vizheh et al. 2023). Research has indicated that this could be via knowledge, incentives, or provider support for the use of contraception (USAID 2013). Our findings demonstrate that the association of CHW engagement is with consistent use, switching between methods, and discontinuation of methods. CHW engagement was associated with initiation of both traditional and modern methods among nonusers, with consistent use and switching to the modern method and discontinuation of the modern method, demonstrating complexity in the trajectories of use. It is possible that CHW engagement enables and motivates women towards contraception use, including use among nonusers, as well as to discontinue use. To assess how CHW engagement is associated with an often used measure of agency—decision‐making—we conducted a descriptive analysis of CHW contact with decision‐making by self, husband, or joint, and found that CHW contact was higher when the decision was made by the woman herself or by her husband, and lower when the decision was made jointly (*p* < 0.0001). Previous research has shown that decision‐making by self and husband has been a strong predictor of contraceptive use, but speaks to different views of agency. These complex findings only underscore the need for more nuanced measures of agency in large‐scale surveys and conducting mediation analyses in studies examining the role of health workers to unpack the direction of influence.

However, whether traditional or modern method of contraception—any choice must reflect women's own contraceptive goals and the agency to achieve those goals; implementers and researchers must not consider only the use of modern contraceptive use as a marker of program success, but the enabling of any form of voice or choice as a viable marker of program success (Bhan and Raj 2021; Jejeebhoy and Sathar 2024; Fabic et al. 2023; Jadhav, Fabic, and MacQuarrie 2025; Dehlendorf et al. 2025). Indeed, reproductive agency is a dynamic process preceded by critical consciousness, which is enabled by awareness and knowledge such as that which a CHW may offer (Raj et al. 2021). In this framing, reproductive agency reflects a cycle of self‐awareness, action, and continued action in the face of barriers towards the pursuit of fertility or FP goals as part of the empowerment process.

While our analysis uses the framing of reproductive agency (Raj et al. 2021) and reproductive justice (Jejeebhoy and Sathar 2024), existing data constrain our ability to advance a person‐centered understanding of the association. In our study, we found that CHW contact and FP discussion during contact were associated with initiation, consistent use, switching and discontinuation, reflecting the need to examine the ways in which CHWs approach and engage with women and couples. It is possible that this engagement facilitates information, self‐efficacy, motivation, and valuable services; this remains a gap in the absence of follow‐ups and a longitudinal understanding of the user journey. This has been indicated in the few studies that show improvements in health‐seeking and male involvement (August et al. 2016; McConnell et al. 2016).

Our study also showed that CHW contact was distributed unevenly. Younger age (15–19 year olds) or zero parity women had the least contact with CHWs, who represented the health system. Their isolation from the health system may be attributed to reproductive control exercised by family members (husbands or in‐laws), lack of freedom of movement, needing permission from others to use contraception, or to make social connections, especially early in marriage (Kumar et al. 2020; Bhan et al. 2022). There is some evidence that health workers or providers may also be influenced by fertility norms and avoid reaching out to lower parity women or engage with reproductive age women after the first childbirth (Sidze et al. 2014). These areas need greater exploration as these have implications for the use of FP services, while underscoring the complex manifestations of power within discussions of choice and agency.

Fourth, in our study, we found that among women who reported recent CHW contact, about one fifth of them reported having discussed FP issues. This might seem reasonable given the diversity of tasks and workload assigned to health workers and their outreach with diverse populations in the community. However, current surveys and implementation research have not adequately captured the nature, tone, and content of health worker interaction, barring provision of information on a contraceptive method and its side effects, and this remains a black box with implications for method choice, use, switching, and discontinuation (Shukla et al. 2020; Diamond‐Smith et al. 2020). Are these discussions informative, supportive, or coercive? Does this outreach depend on women's profiles and parity or factors related to a provider? Are health workers trained to offer information and support to women and refer them in time to the services needed? Do health workers, by commission or omission, promote methods selectively, thereby restricting women's choices for contraception? Understanding the nature of these interactions has important implications for implementation research and FP programs. The content and quality of these encounters and the outreach that promotes them are particularly key to explore in the context of a static method mix in India that lacks new methods of contraception.

Finally, descriptive reasons for switching and discontinuation of methods show a mix of positives and negatives for FP programs. On a positive note, nearly one third of those who switched from traditional contraception to modern methods reported wanting a more effective method, demonstrating accuracy in their knowledge and understanding on effectiveness as well as the success of CHW outreach. Family opposition, especially from husbands, was also an important reason for method discontinuation, showing the control of partners/ husbands over women's FP choices. Among modern method users, specific method‐related complaints remained important reasons for discontinuation of contraception, demonstrating the need for FP service delivery that is more attuned to women's preferences in use, and offering methods that match this choice.

While there has been much to celebrate for FP programs in India and other LMICs with a rise in modern contraceptive availability and options over recent years, there has been some confusion in the FP community on how to interpret the rising use of traditional methods within the former context. From a health policy framing, much of the discussion on traditional methods has been about considering these methods as alternatives to modern methods. In this study, we found that women often use these methods together with modern methods, and the use was associated with contact with a CHW in the past three months. The study raises important questions related to the connectivity of women to their health services and the nature of contraceptive counseling that leads to use or discontinuation.

Research on traditional contraceptive use in LMICs has been growing and provides insight on women's choices within FP programs. Previous studies have investigated the sociodemographic determinants of traditional contraception use (Nketiah‐Amponsah, Ampaw, and Twumasi Baffour 2022; Kundu et al. 2022); this study extends this to understand the association between CHW contact and the type of contraceptives used. Our study is also unique as it uses detailed calendar data to understand switching and discontinuation of methods, providing a more accurate account of use and switching between methods. This is examined within a three‐month time frame, which provides reasonable recall and constrains the association between contact with a health worker and the dynamics of use to this limited period. However, within this time frame, the exact timing of the alignment between the contact and the dynamics of use cannot be established with these data. The three‐month time frame does not guarantee health worker contact prior to changes in contraceptive status, nor does the nature of the analyses presented here allow for conclusions of causality; all findings should thus be understood as associations rather than suggesting a direct causal effect. This approach has advantages in reducing recall bias known to be present in retrospective reporting of contraceptive use, particularly using calendar data, and for women using traditional methods of contraception (Anglewicz et al. 2023; Callahan and Becker 2012). While the three‐month metric has advantages in terms of lower recall bias relative to a longer time frame, it can also be limiting in our understanding of outreach (e.g., women may be in touch with health services, but not in the past three months).

Another limitation of our study is the lack of data on the nature of contact, including the content, tone, or communication dynamics. This has important implications for whether and how contact might influence dynamic contraceptive use. Additionally, reported use of traditional contraception, either directly accessed through the current use measure or as prior three‐month use in the calendar data, does not account for correct or ideal use of the method and may not actually represent effective contraception. Indeed, typical‐use failure rates for withdrawal and rhythm methods are high, and we do not attempt to validate or correct for less‐than‐ideal or imperfect method use as the focus of these analyses was on reported/perceived contraceptive use (Polis et al. 2016). Future research that examines the quality (e.g., consistency, accuracy in timing) of withdrawal and rhythm method use may help add nuance to the understanding of traditional methods in this context, and how CHWs may engage with women to, for example, improve efficacy of these methods.

## CONCLUSION

Our study examined the association between CHW contact and contraceptive use in India. We found that traditional contraception use was high, particularly among married nonpregnant, non‐sterilized women ages 15–49 years in India, and was used singularly as well as concurrently with modern methods. We found FP discussion during CHW contact to be associated with higher uptake of modern and traditional contraception and continued use of modern contraception methods. We also found CHW contact to be associated with recent switching from nonuse to traditional and modern methods and from traditional to modern contraception methods. Our findings demonstrate the need to consider the dynamics of switching and concurrent use, in addition to single method use, in analyses that show traditional methods as complementary to modern contraception. Efforts are also needed to ensure that FP programs consider all contraceptive method choices—not solely modern methods—as potential outcomes of agency and to examine the complex dynamics that guide switching and concurrent use of contraceptive methods.

## CONFLICT OF INTEREST STATEMENT

The authors declare no conflicts of interest.

## ETHICS APPROVAL STATEMENT

This study used NFHS data that were reviewed and approved by the ethical review boards of the IIPS, India, and ICF USA; all other DHS surveys were reviewed and approved by in‐country review boards and ICF USA. This analysis utilized publicly available de‐identified open‐access data from the National Family Health Surveys (globally known as the Demographic and Health Surveys) and received a determination of Not Human Subjects Research from the Institutional Review Board of the University of California, San Diego.

## Supporting information




**Table A1**: Current use of traditional contraception methods across Indian states among non‐pregnant non‐sterilized women in the National Family Health Surveys 2019‐20 (n = 306,037)
**Table A2**: Current use of reversible modern contraception methods across Indian states among non‐pregnant non‐sterilized women in the National Family Health Surveys 2019‐20 (n = 306,037)
**Table A3**. Association between recent contact with health worker and switching or staying consistent with traditional method use among non‐pregnant, non‐sterilized women in the National Family Health Survey – 5 (2019‐21), overall (n = 304,381), among the 9 states with highest quartile of traditional use prevalence (n = 101,855), and among the 9 states with the lowest quartile of traditional use prevalence (n = 53,391)

## Data Availability

This study used publicly available data from the National Family Health Survey (NFHS) – 5 (2019–2021). These data are available from the Measure Demographic and Health Surveys website [https://dhsprogram.com/Data/].

## References

[sifp70052-bib-0001] Ajayi, Anthony Idowu , Oladele Vincent Adeniyi , and Wilson Akpan . 2018. “Use of Traditional and Modern Contraceptives Among Childbearing Women: Findings From a Mixed Methods Study in Two Southwestern Nigerian States.” BMC Public Health 18: 1–9. 10.1186/s12889-018-5522-6 PMC5941455 29739372

[sifp70052-bib-0002] Anglewicz, Philip , Dana Sarnak , Alison Gemmill , and Stan Becker . 2023. “Characteristics Associated With Reliability in Reporting of Contraceptive Use: Assessing the Reliability of the Contraceptive Calendar in Seven Countries.” Studies in Family Planning 54 (1): 17–38. 36715569 10.1111/sifp.12226

[sifp70052-bib-0003] August, Furaha , Andrea B. Pembe , Rose Mpembeni , Pia Axemo , and Elisabeth Darj . 2016. “Community Health Workers Can Improve Male Involvement in Maternal Health: Evidence from Rural Tanzania.” Global Health Action 9 (1): 30064. 26790461 10.3402/gha.v9.30064 PMC4720685

[sifp70052-bib-0004] Bajwa, Sukhwinder Kaur , Sukhminder Jit Singh Bajwa , Gagandeep Kaur Ghai , Kamaljit Singh , and Nirankar Singh . 2012. “Knowledge, Attitudes, Beliefs, and Perception of the North Indian Population toward Adoption of Contraceptive Practices.” Asia Pacific Journal of Public Health 24 (6): 1002–1012. 21807625 10.1177/1010539511411473

[sifp70052-bib-0005] Bationo, Nestor , Patrice Alain Ngangue , Dieudonne Soubeiga , Yacouba Pafadnam , Abibata Fleur Barro , Hermann Pilabre , Ahmed Kabore , Sulpice Adognibo , and Maxime K. Drabo . 2022. “Preferences and Motivations of Women Who Use Traditional Contraceptive Methods to Avoid Pregnancy in Sub‐Saharan Africa: A Systematic Review.” Advances in Sexual Medicine 12 (2) 47–63. https://www.scirp.org/journal/paperinformation.aspx?paperid=116405.

[sifp70052-bib-0006] Berglas, Nancy F. , Katrina Kimport , Aisha Mays , Shelly Kaller , and M. Antonia Biggs . 2021. “‘It's Worked Well for Me’: Young Women's Reasons for Choosing Lower‐Efficacy Contraceptive Methods.” Journal of Pediatric and Adolescent Gynecology 34 (3): 341–347. 33359316 10.1016/j.jpag.2020.12.012 PMC8096642

[sifp70052-bib-0007] Bertrand, Jane T. , John Ross , and Annie L. Glover . 2022. “Declining Yet Persistent Use of Traditional Contraceptive Methods in Low‐and Middle‐Income Countries.” Journal of Biosocial Science 54 (5): 742–759. 34269170 10.1017/S0021932021000341 PMC11753471

[sifp70052-bib-0008] Bhan, Nandita , and Anita Raj . 2021. “From Choice to Agency in Family Planning Services.” The Lancet 398 (10295): 99–101. 10.1016/S0140-6736(21)00990-9 33971154

[sifp70052-bib-0009] Bhan, Nandita , Chhavi Sodhi , Pranita Achyut , Edwin Elizabeth Thomas , Abhishek Gautam , and Anita Raj . 2022. “Mother‐in‐Law's Influence on Family Planning Decision‐Making and Contraceptive Use: A Review of Evidence.” San Diego: Center on Gender Equity and Health and International Center for Research on Women .

[sifp70052-bib-0010] Bradley, Sarah E.K. , William Winfrey , and Trevor Croft . 2015. Contraceptive Use and Perinatal Mortality in the DHS: An Assessment of the Quality and Consistency of Calendars and Histories. Rockville, MD: ICF International.

[sifp70052-bib-0011] Brooks, Mohamad I. , Nicole E. Johns , Anne K. Quinn , Sabrina C. Boyce , Ibrahima A. Fatouma , Alhassane O. Oumarou , Aliou Sani , and Jay G. Silverman . 2019. “Can Community Health Workers Increase Modern Contraceptive Use among Young Married Women? A Cross‐Sectional Study in Rural Niger.” Reproductive Health 16: 1–10. 30909942 10.1186/s12978-019-0701-1 PMC6434879

[sifp70052-bib-0012] Callahan, Rebecca L. , and Stan Becker . 2012. “The Reliability of Calendar Data for Reporting Contraceptive Use: Evidence from Rural Bangladesh.” Studies in Family Planning 43 (3): 213–222. 23185864 10.1111/j.1728-4465.2012.00319.x PMC3628694

[sifp70052-bib-0013] Castro Lopes, Sofia , Deborah Constant , Silvia Fraga , and Jane Harries . 2022. “How Women's Empowerment Influences Fertility‐Related Outcomes and Contraceptive Practices: A Cross‐Sectional Study in Mozambique.” PLoS Global Public Health 2 (9): e0000670. 36962719 10.1371/journal.pgph.0000670 PMC10021614

[sifp70052-bib-0014] Cohen, Nicki , Finou Thérèse Mendy , Jennifer Wesson , Amanda Protti , Carol Cissé , Elhadji Babacar Gueye , Lydia Trupe , Rosii Floreak , Dana Guichon , Karina Lorenzana , and Alison Buttenheim . 2020. “Behavioral Barriers to the Use of Modern Methods of Contraception among Unmarried Youth and Adolescents in Eastern Senegal: A Qualitative Study.” BMC Public Health 20 (1): 1025. 10.1186/s12889-020-09131-4. 32600290 PMC7325306

[sifp70052-bib-0015] Croft, Trevor N. , Courtney K. Allen , and Blake W. Zachary . 2023. Guide to DHS Statistics. Rockville, MD: ICF.

[sifp70052-bib-0016] Dehlendorf, Christine , Karen Hardee , Evelyne Opondo , and Anita Raj . 2025. “Moving Past a Legacy of Controlling Women: Key Frameworks to Center Women and Girls' Choice and Agency in Sexual and Reproductive Health Measurement.” Studies in Family Planning 56 (3): 372–389. 40631486 10.1111/sifp.70027 PMC12501724

[sifp70052-bib-0017] Department of Health & Family Welfare . 2020. “Annual Report 2019–20.” New Delhi: Ministry of Health & Family Welfare, Government of India.

[sifp70052-bib-0018] Diamond‐Smith, Nadia , Claire McDonell , Ananta Basudev Sahu , Kali Prasad Roy , and Katie Giessler . 2020. “A Mixed‐Methods Evaluation of the Impact of a Person‐Centered Family Planning Intervention for Community Health Workers on Family Planning Outcomes in India.” BMC Health Services Research 20: 1–12. 10.1186/s12913-020-05995-9 PMC7733295 33308230

[sifp70052-bib-0019] Dixit, A. , N. E. Johns , M. Ghule , M. Battala , S. Begum , J. Yore , N. Saggurti , J. G. Silverman , E. Reed , T. Benmarhnia , S. Averbach , and A. Raj . 2021. “Male‐Female Concordance in Reported Involvement of Women in Contraceptive Decision‐Making and Its Association With Modern Contraceptive Use Among Couples in Rural Maharashtra, India.” Reproductive Health 18 (1): 139. 10.1186/s12978-021-01187-8. 34193214 PMC8244175

[sifp70052-bib-0020] Ewerling, F. , L. McDougal , A. Raj , L. Z. Ferreira , C. Blumenberg , D. Parmar , and A. J. D. Barros . 2021. “Modern Contraceptive Use among Women in Need of Family Planning in India: An Analysis of the Inequalities Related to the Mix of Methods Used.” Reproductive Health 18 (1): 173. 10.1186/s12978-021-01220-w. 34419083 PMC8379729

[sifp70052-bib-0021] Fabic, Madeleine Short , Lotus McDougal , Anita Raj , and Apoorva Jadhav . 2023. “Is the Decision Not to Use Contraception an Indicator of Reproductive Agency?” Studies in Family Planning 54 (1): 95–117. 36790883 10.1111/sifp.12235

[sifp70052-bib-0022] Filmer, Deon , and Lant H. Pritchett . 2001. “Estimating Wealth Effects Without Expenditure Data—or Tears: An Application to Educational Enrollments in States of India.” Demography 38 (1): 115–132. 10.1353/dem.2001.0003. 11227840

[sifp70052-bib-0023] Gebreselassie, Tesfayi , Kristin Bietsch , Sarah Staveteig , and Thomas Pullum . 2017. “Trends, Determinants, and Dynamics of Traditional Contraceptive Method Use.” Rockville, MD: ICF.

[sifp70052-bib-0024] Gupte, Prajakta R. 2017. “India: ‘The Emergency’ and the Politics of Mass Sterilization.” Education About Asia 22 (3): 40–44.

[sifp70052-bib-0025] Halli, Shiva S. , Mohd Tauheed Alam , Antony Joseph , Ravi Prakash , Shajy Isac , Marissa Becker , Preeti Anand , N Vasanthakumar , B.M. Ramesh , and James Blanchard . 2023. “Declining Fertility and Increasing Use of Traditional Methods of Family Planning: A Paradox in Uttar Pradesh, India?” Journal of Biosocial Science 55 (2): 224–237. 35249572 10.1017/S0021932022000086

[sifp70052-bib-0026] Howe, Laura D. , James R. Hargreaves , and Sharon R.A. Huttly . 2008. “Issues in the Construction of Wealth Indices for the Measurement of Socio‐Economic Position in Low‐Income Countries.” Emerging Themes in Epidemiology 5: 1–14. 18234082 10.1186/1742-7622-5-3 PMC2248177

[sifp70052-bib-0027] ICF International Inc . “MEASURE DHS STATcompiler.” Rockville, MD: ICF International. Accessed January 9. www.statcompiler.com.

[sifp70052-bib-0028] Jadhav, Apoorva , Madeleine Short Fabic , and Kerry MacQuarrie . 2025. “How It Was and How It Should Be: Moving Toward a Better Measurement of Contraceptive Prevalence Among Unmarried Women.” Studies in Family Planning 56 (3): 454–469. 40624791 10.1111/sifp.70028

[sifp70052-bib-0029] James‐Hawkins, Laurie , Courtney Peters , Kristin VanderEnde , Lauren Bardin , and Kathryn M. Yount . 2018. “Women's Agency and Its Relationship to Current Contraceptive Use in Lower‐and Middle‐Income Countries: A Systematic Review of the Literature.” Global Public Health 13 (7): 843–858. 27690750 10.1080/17441692.2016.1239270

[sifp70052-bib-0030] Jejeebhoy, Shireen J. , and Zeba Sathar . 2024. “Revisiting Women's Empowerment and Contraception.” Population and Development Review 50 (S2): 597–623.

[sifp70052-bib-0031] Kalyesubula, Robert , Jessica Mitter Pardo , Stephanie Yeh , Richard Munana , Ivan Weswa , Joseph Adducci , Faith Nassali , Mennen Tefferi , John Mundaka , and Sahai Burrowes . 2021. “Youths' Perceptions of Community Health Workers' Delivery of Family Planning Services: A Cross‐Sectional, Mixed‐Methods Study in Nakaseke District, Uganda.” BMC Public Health 21: 1–15. 33827502 10.1186/s12889-021-10695-y PMC8028711

[sifp70052-bib-0032] Kane, Sumit , Anjali Radkar , Mukta Gadgil , and Barbara McPake . 2020. “Community Health Workers as Influential Health System Actors and Not ‘Just Another Pair of Hands’.” International Journal of Health Policy and Management 10 (8): 465. 10.34172/ijhpm.2020.58 PMC9056200 32610755

[sifp70052-bib-0033] Kibira, Simon P.S. , Celia Karp , Shannon N. Wood , Selamawit Desta , Hadiza Galadanci , Fredrick E. Makumbi , Elizabeth Omoluabi , Solomon Shiferaw , Assefa Seme , and Amy Tsui . 2020. “Covert Use of Contraception in Three Sub‐Saharan African countries: a qualitative exploration of motivations and challenges.” BMC Public Health 20 (1): 865. 32503485 10.1186/s12889-020-08977-y PMC7275340

[sifp70052-bib-0034] Kumar, Abhishek , Anrudh K. Jain , Faujdar Ram , Rajib Acharya , Ankita Shukla , Arupendra Mozumdar , and Niranjan Saggurti . 2020. “Health Workers' Outreach and Intention to Use Contraceptives among Married Women in India.” BMC Public Health 20: 1–9. 32605622 10.1186/s12889-020-09061-1 PMC7329531

[sifp70052-bib-0035] Kundu, Satyajit , Subarna Kundu , Md. Ashfikur Rahman , Humayun Kabir , Md. Hasan Al Banna , Saurav Basu , Hasan Mahmud Reza , and Ahmed Hossain . 2022. “Prevalence and Determinants of Contraceptive Method Use among Bangladeshi Women of Reproductive Age: A Multilevel Multinomial Analysis.” BMC Public Health 22 (1): 2357. 36526989 10.1186/s12889-022-14857-4 PMC9756620

[sifp70052-bib-0036] Marquez, Maria Paz , Maria Midea Kabamalan , and Elma Laguna . 2018. “Traditional and Modern Contraceptive Method Use in the Philippines: Trends and Determinants 2003–2013.” Studies in Family Planning 49 (2): 95–113. 29665078 10.1111/sifp.12051

[sifp70052-bib-0037] McConnell, Margaret , Allison Ettenger , Claire Watt Rothschild , Faith Muigai , and Jessica Cohen . 2016. “Can a Community Health Worker Administered Postnatal Checklist Increase Health‐Seeking Behaviors and Knowledge?: Evidence from a Randomized Trial with a Private Maternity Facility in Kiambu County, Kenya.” BMC Pregnancy and Childbirth 16: 1–19. 27260500 10.1186/s12884-016-0914-z PMC4893209

[sifp70052-bib-0038] Mejía‐Guevara, Iván , Beniamino Cislaghi , and Gary L. Darmstadt . 2021. “Men's Attitude towards Contraception and Sexuality, Women's Empowerment, and Demand Satisfied for Family Planning in India.” Frontiers in Sociology 6: 689980. 34977228 10.3389/fsoc.2021.689980 PMC8717326

[sifp70052-bib-0039] Namasivayam, Vasanthakumar , Bidyadhar Dehury , Ravi Prakash , Marissa Becker , Preeti Anand , Ashish Mishra , Shreya Singhal , Shivalingappa Halli , James Blanchard , and Dean Spears . 2023. “Understanding the Rise in Traditional Contraceptive Methods Use in Uttar Pradesh, India.” Reproductive Health 20 (1): 8. 36609308 10.1186/s12978-022-01547-y PMC9817250

[sifp70052-bib-0040] Nketiah‐Amponsah, Edward , Samuel Ampaw , and Priscilla Twumasi Baffour . 2022. “Socioeconomic Determinants of Use and Choice of Modern Contraceptive Methods in Ghana.” Tropical Medicine and Health 50 (1): 33. 35581604 10.1186/s41182-022-00424-5 PMC9116020

[sifp70052-bib-0041] Poirier, Mathieu J.P. , Karen A. Grépin , and Michel Grignon . 2020. “Approaches and Alternatives to the Wealth Index to Measure Socioeconomic Status Using Survey Data: A Critical Interpretive Synthesis.” Social Indicators Research 148 (1): 1–46.

[sifp70052-bib-0042] Polis, Chelsea B. , Sarah E.K. Bradley , Akinrinola Bankole , Tsuyoshi Onda , Trevor Croft , and Susheela Singh . 2016. “Typical‐Use Contraceptive Failure Rates in 43 Countries with Demographic and Health Survey Data: Summary of a Detailed Report.” Contraception 94 (1): 11–17. 27018154 10.1016/j.contraception.2016.03.011 PMC4970461

[sifp70052-bib-0043] Polis, Chelsea B. , Easmon Otupiri , Suzanne O. Bell , and Roderick Larsen‐Reindorf . 2021. “Use of Fertility Awareness‐Based Methods for Pregnancy Prevention Among Ghanaian Women: A Nationally Representative Cross‐Sectional Survey.” Global Health: Science and Practice 9 (2): 318–331. 34234024 10.9745/GHSP-D-20-00601 PMC8324203

[sifp70052-bib-0044] Rabiu, Ayyuba. 2018. “The Role of Traditional Contraceptive Methods in Family Planning among Women Attending Primary Health Care Centers in Kano.” Annals of African medicine 17 (4): 189–195. 30588932 10.4103/aam.aam_60_17 PMC6330776

[sifp70052-bib-0045] Raj, A. , A.K. Dey , R. Lundgren , and EMERGE Team . 2021. A Conceptual Framework for Measuring Women's Empowerment. San Diego, CA: Center on Gender Health and Equity, University of California San Diego. https://emerge.ucsd.edu/wp‐content/uploads/2021/04/emergeconceptualframework‐to‐measure‐empowerment.pdf

[sifp70052-bib-0046] Rajkhowa, Pallavi , and Matin Qaim . 2022. “Mobile Phones, Women's Physical Mobility, and Contraceptive Use in India.” Social Science & Medicine 305: 115074. 35665688 10.1016/j.socscimed.2022.115074

[sifp70052-bib-0047] Ram, Faujdar , Chander Shekhar , and Biswabandita Chowdhury . 2014. “Use of Traditional Contraceptive Methods in India & Its Socio‐demographic Determinants.” Indian Journal of Medical Research 140 (Suppl. 1): S17‐S28. 25673538 PMC4345748

[sifp70052-bib-0048] Rossier, Clémentine , and Jamaica Corker . 2017. “Contemporary Use of Traditional Contraception in Sub‐Saharan Africa.” Population and Development Review 43 (Suppl. 1): 192. 29307923 10.1111/padr.12008 PMC5749414

[sifp70052-bib-0049] Rutenberg, Naomi , and Susan Cotts Watkins . 1997. “The Buzz Outside the Clinics: Conversations and Contraception in Nyanza Province, Kenya.” *Studies in Family Planning* 28, No. 4: 290–307. 9431650

[sifp70052-bib-0050] Rutstein, Shea O. 2015. “Steps to Constructing the New DHS Wealth Index.” Rockville, MD: ICF International .

[sifp70052-bib-0051] Schaaf, Marta , Caitlin Warthin , Lynn Freedman , and Stephanie M. Topp . 2020. “The Community Health Worker as Service Extender, Cultural Broker and Social Change Agent: A Critical Interpretive Synthesis of Roles, Intent and Accountability.” BMJ Global Health 5 (6): e002296. 10.1136/bmjgh-2020-002296 PMC7299037 32546585

[sifp70052-bib-0052] Scott, Valerie K. , Lindsey B. Gottschalk , Kelsey Q. Wright , Claire Twose , Meghan A. Bohren , Megan E Schmitt , and Nuriye Ortayli . 2015. “Community Health Workers' Provision of Family Planning Services in Low‐and Middle‐Income Countries: A Systematic Review of Effectiveness.” Studies in Family Planning 46 (3): 241–261. 26347089 10.1111/j.1728-4465.2015.00028.x

[sifp70052-bib-0053] Sharma, Mukesh Kumar , Emily Das , Hitesh Sahni , Jessica Mirano , Kate Graham , Abhishek Kumar , and Clea Finkle . 2024. “Engaging Community Health Workers to Enhance Modern Contraceptive Uptake among Young First‐Time Parents in Five Cities of Uttar Pradesh.” Global Health: Science and Practice 12 (Suppl. 2): e2200170. 38575360 10.9745/GHSP-D-22-00170 PMC11111106

[sifp70052-bib-0054] Shea, Amanda A. , Meghana Kulkarni , Jonathan Thornburg , Cécile Ventola , Erin Walker , and Virginia J. Vitzthum . 2024. “A Bother or a Benefit? How Contraceptive Users Balance the Trade‐Offs between Preferred Menstrual Bleeding Patterns and Preferred Contraceptive Methods in India, South Africa, and the United States.” Women's Reproductive Health 11 (2): 343–382.

[sifp70052-bib-0055] Shukla, Ankita , Rajib Acharya , Abhishek Kumar , Arupendra Mozumdar , Kumudha Aruldas , and Niranjan Saggurti . 2020. “Client‐Provider Interaction: Understanding Client Experience with Family Planning Service Providers through the Mystery Client Approach in India.” Sexual and Reproductive Health Matters 28 (1): 1822492. 33054696 10.1080/26410397.2020.1822492 PMC7566859

[sifp70052-bib-0056] Sidze, Estelle M. , Solène Lardoux , Ilene S. Speizer , Cheikh M. Faye , Michael M. Mutua , and Fandi Badji . 2014. “Young Women Access and Use of Contraception: The Role of Providers' Restrictions in Urban Senegal.” International Perspectives on Sexual and Reproductive Health 40 (4): 176. 25565345 10.1363/4017614 PMC6652199

[sifp70052-bib-0057] Soin, Komal S. , Ping Teresa Yeh , Mary E. Gaffield , Christina Ge , and Caitlin E. Kennedy . 2022. “Health Workers' Values and Preferences Regarding Contraceptive Methods Globally: A Systematic Review.” Contraception 111: 61–70. 35526598 10.1016/j.contraception.2022.04.012 PMC9233149

[sifp70052-bib-0058] UNDESA . 2022. “World Family Planning 2022: Meeting the Changing Needs for Family Planning: Contraceptive Use by Age and Method.” New York: UN Department of Economic and Social Affairs, Population Division.

[sifp70052-bib-0059] USAID . 2013. “High‐Impact practices in family planning (HIP).” Family Planning and Immunization Integration: Reaching Postpartum Women With Family Planning Services. USAID. Washington, DC.

[sifp70052-bib-0060] Vizheh, Maryam , Frances Rapport , Jeffrey Braithwaite , and Yvonne Zurynski . 2023. “The Impact of Women's Agency on Accessing and Using Maternal Healthcare Services: A Systematic Review and Meta‐Analysis.” International Journal of Environmental Research and Public Health 20 (5): 3966. 36900977 10.3390/ijerph20053966 PMC10002172

[sifp70052-bib-0061] Wagner, Abram L. , Julia M. Porth , Deepti Bettampadi , and Matthew L. Boulton . 2018. “Have Community Health Workers Increased the Delivery of Maternal and Child Healthcare in India?” Journal of Public Health 40 (2): e164‐e170. 28985399 10.1093/pubmed/fdx087

[sifp70052-bib-0062] WHO . 2022. Family Planning: A Global Handbook for Providers (2022 update). Baltimore: CCP and WHO.

[sifp70052-bib-0063] Wolff, Brent , Ann K. Blanc , and John Ssekamatte‐Ssebuliba . 2000. “The Role of Couple Negotiation in Unmet Need for Contraception and the Decision to Stop Childbearing in Uganda.” Studies in Family Planning 31 (2): 124–137. 10907278 10.1111/j.1728-4465.2000.00124.x

