# Supplementary material for: Traditional or Modern Contraception? Association Between Health Worker Contact and Contraceptive Choice in India: Findings From NFHS 2019–2021
Source: Stud Fam Plann. 2026 Apr 7;57(2):209–33. doi: 10.1111/sifp.70052 (PMC13275035; doi:10.1111/sifp.70052)
Supplement: Supplementary file 1 — Table A1: Current use of traditional contraception methods across Indian states among non‐pregnant non‐sterilized women in the National Family Health Surveys 2019‐20 (n = 306,037) Table A2: Current use of reversible modern contraception methods across Indian states among non‐pregnant non‐sterilized women in the National Family Health Surveys 2019‐20 (n = 306,037) Table A3. Association between recent contact with health worker and switching or staying consistent with traditional method use among non‐pregnant, non‐sterilized women in the National Family Health Survey – 5 (2019‐21), overall (n = 304,381), among the 9 states with highest quartile of traditional use prevalence (n = 101,855), and among the 9 states with the lowest quartile of traditional use prevalence (n = 53,391) [file SIFP-57-209-s001.docx]

***ONLINE SUPPLEMENTARY MATERIAL***

*Table A1:* *Current use of traditional contraception methods across Indian states among non-pregnant non-sterilized women in the National Family Health Surveys 2019-20* (n=306,037)

| **Indian States** | **Percent (%) reported use of traditional contraception** |
| --- | --- |
| *All India* | *22.1%* |
| Mizoram | 0.6% |
| Andhra Pradesh | 1.1% |
| Telangana | 5.0% |
| Karnataka | 5.4% |
| Ladakh | 5.7% |
| Maharashtra | 6.6% |
| Meghalaya | 8.1% |
| Tamil Nadu | 9.4% |
| Puducherry | 10.1% |
| Jammu & Kashmir | 16.2% |
| Dadra and Nagar Haveli | 16.7% |
| Madhya Pradesh | 16.8% |
| Goa | 17.1% |
| Chhattisgarh | 17.2% |
| Kerala | 17.9% |
| Andaman-Nicobar | 18.3% |
| Nagaland | 18.9% |
| Arunachal Pradesh | 19.4% |
| Bihar | 21.8% |
| Assam | 22.4% |
| Himachal Pradesh | 22.5% |
| Sikkim | 22.7% |
| Gujarat | 22.9% |
| Jharkhand | 24.1% |
| Haryana | 24.8% |
| Rajasthan | 24.9% |
| West Bengal | 25.6% |
| Uttar Pradesh | 28.9% |
| Uttarakhand | 29.9% |
| Punjab | 31.6% |
| Lakshadweep | 31.6% |
| NCT of Delhi | 31.8% |
| Tripura | 32.7% |
| Chandigarh | 35.6% |
| Odisha | 41.4% |
| Manipur | 51.7% |

*Table A2:* *Current use of reversible modern contraception methods across Indian states among non-pregnant non-sterilized women in the National Family Health Surveys 2019-20* (n=306,037)

| **Indian States** | **% reported use of reversible modern contraception** |
| --- | --- |
| *All India* | *32.5%* |
| NCT of Delhi | 51.1% |
| Sikkim | 48.9% |
| Chandigarh | 47.2% |
| West Bengal | 47.0% |
| Uttarakhand | 46.1% |
| Tripura | 45.3% |
| Haryana | 45.2% |
| Goa | 45.1% |
| J & K | 45.1% |
| Himachal Pradesh | 44.0% |
| Ladakh | 42.6% |
| Assam | 42.5% |
| Nagaland | 40.7% |
| Arunachal Pradesh | 38.1% |
| Rajasthan | 37.7% |
| Punjab | 37.5% |
| Uttar Pradesh | 35.8% |
| Dadra and Nagar Haveli | 33.5% |
| Andaman-Nicobar | 31.9% |
| Madhya Pradesh | 31.8% |
| Maharashtra | 31.4% |
| Odisha | 30.8% |
| Chhattisgarh | 30.1% |
| Gujarat | 29.7% |
| Karnataka | 28.1% |
| Mizoram | 22.0% |
| Jharkhand | 21.2% |
| Puducherry | 20.4% |
| Meghalaya | 20.0% |
| Tamil Nadu | 19.9% |
| Bihar | 16.8% |
| Manipur | 16.2% |
| Telangana | 14.1% |
| Kerala | 12.7% |
| Lakshadweep | 12.7% |
| Andhra Pradesh | 4.6% |

*Table A3. Association between recent contact with health worker and switching or staying consistent with traditional method use among non-pregnant, non-sterilized women in the National Family Health Survey – 5 (2019-21), overall (n=304,381), among the 9 states with highest quartile of traditional use prevalence (n=101,855), and among the 9 states with the lowest quartile of traditional use prevalence (n=53,391)*

|  | **Non-use, traditional discontinuation** | **Non-use, modern discontinuation** | **Traditional use, consistent** | **Modern use, consistent** | **Traditional use, initiated from non-use** | **Modern use, initiated from non-use** | **Traditional use, switched from modern method** | **Modern use, switched from traditional method** |
| --- | --- | --- | --- | --- | --- | --- | --- | --- |
| Overall | 1.09  [0.97-1.24]  p=0.14 | **1.42**  **[1.26-1.59]**  **p<0.001** | **0.86**  **[0.82-0.89]**  **p<0.001** | **1.18**  **[1.14-1.22]**  **p<0.001** | **1.67**  **[1.51-1.85]**  **p<0.001** | **2.16**  **[2.01-2.33]**  **p<0.001** | 1.18  [0.86-1.63]  p=0.30 | **1.92**  **[1.36-2.73]**  **p<0.001** |
| Highest quartile of traditional use prevalence states* | 1.10  [0.92-1.31]  p=0.28 | **1.47**  **[1.24-1.74]**  **p<0.001** | **0.71**  **[0.67-0.75]**  **p<0.001** | 1.01  [0.96-1.07]  p=0.68 | **1.56**  **[1.35-1.80]**  **p<0.001** | **2.04**  **[1.82-2.27]**  **p<0.001** | 1.03  [0.65-1.62]  p=0.90 | **1.60**  **[1.15-2.22]**  **p=0.005** |
| Lowest quartile of traditional use prevalence states** | 1.30  [0.71-2.36]  p=0.40 | **1.54**  **[1.14-2.08]**  **p=0.005** | 1.13  [0.94-1.37]  p=0.20 | **1.46**  **[1.32-1.61]**  **p<0.001** | **2.15**  **[1.26-3.69]**  **p=0.005** | **1.92**  **[1.54-2.40]**  **p<0.001** | --*** | **--***** |

*Highest traditional method use prevalence states & union territories: Manipur, Odisha, Chandigarh, Tripura, NCT of Delhi, Punjab, Lakshadweep, Uttarakhand, Uttar Pradesh

**Lowest traditional method use prevalence states & union territories: Puducherry, Tamil Nadu, Meghalaya, Maharashtra, Ladakh, Karnataka, Telangana, Andhra Pradesh, Mizoram

***Number of women switching method types in past 3 months among low-prevalence traditional method use states was too small to estimate these outcome categories [n=7 women switching from modern to traditional, n=16 switching from traditional to modern].
